# Isoform D of vascular endothelial growth factor in systemic capillary leak syndrome: a case report

**DOI:** 10.1186/s13256-016-0894-7

**Published:** 2016-05-24

**Authors:** Soushi Ibata, Tsutomu Sato, Kohichi Takada, Ayumi Tatekoshi, Akari Hashimoto, Yusuke Kamihara, Wataru Jomen, Hiroto Horiguchi, Kaoru Ono, Kazuyuki Murase, Satoshi Iyama, Koji Miyanishi, Yasushi Sato, Rishu Takimoto, Masayoshi Kobune, Junji Kato

**Affiliations:** Department of Medical Oncology and Hematology, School of Medicine, Sapporo Medical University, South-1, West-16, Chuo-ku, Sapporo, Japan

## Abstract

**Background:**

Systemic capillary leak syndrome is a rare condition characterized by episodic attacks of hypovolemia due to systemic capillary hyperpermeability, which results in profound hypotension and edema. Although the implication of vascular endothelial growth factor, angiopoietin-2, and C-X-C motif chemokine 10 has been suggested, the pathogenesis of systemic capillary leak syndrome remains unclear. In this report, we describe a case of systemic capillary leak syndrome in which serum isoform D of vascular endothelial growth factor was elevated. To the best of our knowledge, this is the first reported case of systemic capillary leak syndrome in which isoform D of vascular endothelial growth factor is suggested as the plausible biomarker.

**Case presentation:**

A 41-year-old Japanese man was transferred to our emergency department. He was hypotensive, tachycardic, and edematous over the trunk and all four limbs. He received aggressive intravenous fluid therapy and underwent fasciotomy of the right forearm to prevent muscle necrosis. A diagnosis of systemic capillary leak syndrome was suspected. The presence of serum monoclonal immunoglobulin G and κ light chain supported this diagnosis. Prevention of hypotensive crises was unsuccessfully attempted with theophylline, intravenous immunoglobulin, high-dose dexamethasone, bortezomib, melphalan, and prednisolone; however, the patient’s attacks dramatically disappeared after the introduction of thalidomide. The serum of the patient was stored soon after the onset of hypotensive crisis and analyzed to profile possible mediators responsible for the capillary leak. The concentration of vascular endothelial growth factor, angiopoietin-2, and C-X-C motif chemokine 10 were all within normal ranges. Meanwhile, we found that isoform D of vascular endothelial growth factor was elevated, which was normalized after the introduction of thalidomide.

**Conclusions:**

In our patient, isoform D of vascular endothelial growth factor (instead of vascular endothelial growth factor) may have been a causative factor of hypotensive crises, since isoform D contributes to vascular endothelial growth factor receptor-2 signaling, which is the major mediator of the permeability-enhancing effects of vascular endothelial growth factor. We suggest the measurement of isoform D of vascular endothelial growth factor in patients with systemic capillary leak syndrome in whose serum vascular endothelial growth factor is not elevated.

## Background

Systemic capillary leak syndrome (SCLS) is a rare condition characterized by episodic attacks of hypovolemia due to systemic capillary hyperpermeability, which results in profound hypotension and edema. Acute attacks of SCLS usually develop within a few hours and resolve within 1–4 days. The stereotypic laboratory abnormalities during acute attack are hemoconcentration (elevated hematocrit) and hypoalbuminemia. The presence of serum M protein during the quiescent phase between the attacks is a notable common feature. Complications such as acute renal failure, rhabdomyolysis, arrhythmia, pericardial effusion, pleural effusion, deep venous thrombosis, and ischemic stroke during the attacks have been reported [1, 2]. These complications can be directly related to death, and reported survival rates are 89% at 1 year and 73% at 5 years [1]. Although the implication of vascular endothelial growth factor (VEGF), angiopoietin-2 (Ang-2), and C-X-C motif chemokine 10 (CXCL10) has been suggested [3–6], the pathogenesis of SCLS remains unclear.

Many agents have been tried sporadically with varying degrees of effectiveness, including corticosteroids, intravenous immunoglobulin (IVIG), antihistamines, plasmapheresis, and β_2_-agonists (aminophylline, theophylline, or terbutaline) [2]. The anti-VEGF monoclonal antibody bevacizumab has been used successfully in one patient [7]; however, the same anti-VEGF therapy was used in another patient without success [3]. Thalidomide that targets an abnormal plasma cell population or its secreted products has been used in some patients, with improvement in symptoms achieved [1, 8].

In this report, we present a case of a man with SCLS refractory to corticosteroids, theophylline, IVIG, bortezomib, melphalan, and prednisolone, whose attacks were dramatically prevented by the introduction of thalidomide. We also demonstrate the elevated level of isoform D of VEGF (VEGF-D) in this patient’s serum soon after the hypotensive crisis. To the best of our knowledge, this is the first reported case of SCLS in which VEGF-D is suggested as the plausible biomarker.

## Case presentation

A 41-year-old Japanese man was transferred to our emergency department in October 2012 with complaints of headache and nausea. He was hypotensive (98/57 mmHg), tachycardic (120–140 beats/minute), and edematous over the trunk and all four limbs. He had experienced the same episode 9 months previously. That first attack had been complicated by stroke, which caused right hemiplegia. Laboratory studies demonstrated elevated levels of hemoglobin (18.6 g/dl) and hematocrit (53.5%), as well as hypoalbuminemia (2.2 g/dl). To keep his systolic blood pressure from dropping below 60 mmHg, he was given aggressive intravenous fluid therapy (500–1000 ml/hour). Following hydration for more than 12 hours, he complained of severe pain in his right forearm. His forearm compartments were tense and swollen. He underwent fasciotomy of the right forearm to prevent muscle necrosis. A volar longitudinal incision was made. The patient’s hypotension lasted for 2 days, and his symptoms reversed quickly with massive diuresis. One week after fasciotomy, the wound was closed.

In view of these clinical features, a diagnosis of SCLS was suspected. Serum monoclonal immunoglobulin G (IgG) and κ light chain was detected by immunofixation electrophoresis, supporting this diagnosis. The patient’s IgG level was 1143 mg/dl. A bone marrow study revealed 1.0 % of plasma cells. These findings were consistent with a monoclonal gammopathy of undetermined significance. The clinical suspicion of angioedema was not high, because the patient’s complement levels, such as C4 and C1q, were all normal.

**Fig. 1:**
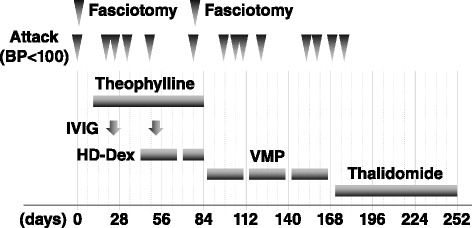
Clinical course of our patient. *BP* blood pressure, *IVIG* intravenous immunoglobulin, *HD-Dex* high-dose dexamethasone, *VMP* bortezomib, melphalan, and prednisolone

Prevention of these crises was attempted with theophylline (400 mg/day), IVIG (2 g/kg/day for 1 day, repeated every 4 weeks), and high-dose dexamethasone (40 mg on days 1–4, 9–12, and 17–20, repeated every 4 weeks); however, five additional episodes of hypotension occurred in the subsequent 3 months after hospitalization (Fig. 1). The last one was so severe that it required repeat fasciotomy of the right forearm. Over the next 3 months, seven episodes ensued despite VMP (bortezomib, melphalan, and prednisolone) therapy, which consisted of bortezomib (1.3 mg/m^2^) on days 1, 8, 15, and 22 with melphalan (9 mg/m^2^) and prednisolone (60 mg/m^2^) on days 1–4. Then, daily oral thalidomide (100 mg) was started. Surprisingly, the patient had no further episodes for 2 years, except for one episode soon after the initiation of thalidomide. Notably, his monoclonal IgG κ protein remains clearly detectable, despite the complete disappearance of his hypotensive crises.

The serum of the patient was stored on the 50th hospital day, immediately after the onset of the 5th hypotensive crisis, to profile possible mediators responsible for the capillary leak. The second sample was serum obtained on the 204th hospital day, when the patient’s hypotensive crisis had disappeared after the introduction of thalidomide. The cytokine levels in the patient’s serum were measured using Quantikine enzyme-linked immunosorbent assay kits (R&D Systems, Minneapolis, MN, USA). Our institutional review board approved the study protocol, and written informed consent was obtained from the patient.

First, we focused on VEGF, Ang-2, and CXCL10, since the possible involvement of these cytokines in SCLS has been indicated in some reports [3–6]. The results presented in Table 1 show that the concentrations of VEGF, Ang-2, and CXCL10 were all within the normal range at each measurement point. Then, we analyzed VEGF isoforms VEGF-C and VEGF-D. The results were that the concentration of VEGF-D on the 50th day was 1042 ng/ml, which exceeded the normal range (135–642 ng/ml). In the serum obtained on the 204th day as the negative control, the VEGF-D level was normal. The concentrations of VEGF-C did not exceed the normal range on either the 50th or the 204th day. Supplemental examinations of four more cytokines—interleukin (IL)-2, IL-4, IL-6, and IL-11—were performed; however, none of them was detected.

**Table 1:** Cytokine levels in the patient’s serum

		Our patient	
Cytokines	Healthy volunteers^a^, mean (range) (*n*)	Day 50	Day 204
VEGF	220 (62–707) (*n* = 37)	97	221
Ang-2	2494 (1065–8907) (*n* = 60)	2266	1899
CXCL10	89 (38–361) (*n* = 60)	251	312
VEGF-C	4847 (2459–6651) (*n* = 30)	2133	4100
VEGF-D	297 (135–642) (*n* = 60)	1042	403

*VEGF* vascular endothelial growth factor, *Ang-2* angiopoietin 2, *CXCL10* C-X-C motif chemokine 10, *VEGF-C* vascular endothelial growth factor-C, *VEGD-D* vascular endothelial growth factor-D

Values are given in picograms per milliliter.

^a^The data indicated in the manufacturer’s written instructions

## Discussion

The etiology of SCLS has not been clarified. One possibility is a monoclonal immunoglobulin often detected in the serum of patients. The clonal plasma cells may produce a toxic monoclonal immunoglobulin that causes pathology by aggregating and depositing in tissue [9]. Although purified monoclonal immunoglobulin from one patient with SCLS failed to bind cultured endothelial cells [3], we speculate that monoclonal immunoglobulin should have an etiological function in some patients with SCLS, because sometimes IVIG is quite effective as a prophylactic therapy for hypotensive episodes [1]. IVIG may neutralize pathogenic monoclonal immunoglobulin, which is a widely accepted theory in treatment of immune thrombocytopenic purpura. Although monoclonal immunoglobulin was clearly detected after the disappearance of the hypotensive crises by the introduction of thalidomide, the possible involvement of monoclonal immunoglobulin as an etiology in our patient could not be ruled out.

Another plausible etiology is the soluble mediators. Recently, three patients with SCLS were reported to have high levels of plasma VEGF during acute episodes, which decreased with symptom resolution [3, 5]. Further, Xie *et al*. examined VEGF and Ang-2 in 20 patients with SCLS and found that these were elevated in episodic SCLS sera but not in remission sera [4]. Xie *et al*. also reported recently that CXCL10 was significantly increased at baseline and in acute SCLS sera relative to controls [6]. Meanwhile, in episodic sera of our patient, not VEGF but its D isoform, VEGF-D, was detected. VEGF-D contributes to VEGF receptor (VEGFR)-2 signaling, which is the major mediator of the mitogenic, angiogenic, and permeability-enhancing effects of VEGF [10]. Thus, VEGF-D, instead of VEGF, may have been a causative mediator of hypotensive crises in our patient. Further, VEGF-D signals through not only VEGFR-2 but also VEFGR-3 (Flt-4). VEGF-D is expressed in the vascular endothelium. VEFGR-3 is expressed predominantly in lymphatic endothelial cells and regulates lymphangiogenesis. VEGF-D has also been implicated in lymphedema. We speculate that VEGF-D may be involved in patients with SCLS whose serum VEGF is not elevated [11] or in patients for whom anti-VEGF antibody bevacizumab is not effective [3], since bevacizumab recognizes VEGF but not VEGF-D [12].

As this report is the first, to the best of our knowledge, that names VEGF-D as a causative mediator, further evaluation in more patients is necessary. Measuring VEGF-D in serum soon after the hypotensive episode might be suggested in some patients with SCLS whose serum VEGF, Ang-2, or CXCL-10 is not high.

As one more plausible etiology, we add the hypersensitivity of VEGFR-2. We speculate that not only VEGF concentration but also VEGFR-2 sensitivity regulates the onset of SCLS, since patients with multiple myeloma often have extremely elevated levels of serum VEGF concentration [13] without any symptoms of SCLS. Both congenital and acquired factors are able to contribute to the hypersensitivity of VEGFR-2. Genome-wide single-nucleotide polymorphism (SNP) analysis of patients with SCLS demonstrated that several SNPs were detected in β-transducin repeat containing E3 ubiquitin protein ligase and dysferlin, which could regulate VEGFR2 signaling [14]. Further examination in these areas is needed.

There remains a problem regarding how thalidomide controlled our patient’s VEGF-D-induced hypotensive crises. Since thalidomide as a single agent achieves only approximately 30% of overall response, with a complete response rate probably in the 10% to at best 15% range even in patients with proliferative myeloma, the eradication of clonal plasma cells with thalidomide in our patient could not be expected. A useful reference is a report by Gupta *et al*., who demonstrated that thalidomide suppressed the production of VEGF from myeloma cells and bone marrow stromal cells [15]. Also, in our patient, thalidomide may have suppressed VEGF-D production from clonal plasma cells or bone marrow stromal cells.

There have been two reports presenting a total of four patients with SCLS treated effectively with thalidomide [1, 8]. Although we have no objection to the opinion of Gousseff *et al*. that β_2_-agonists and IVIG provide prophylaxis as the first-line therapy [8], there must be a certain group of patients with SCLS who could be treated effectively with thalidomide.

## Conclusions

We present a case of SCLS with elevated serum VEGF-D concentration whose hypotensive crises were well-controlled with thalidomide.

## Consent

Written informed consent was obtained from the patient for publication of this case report and any accompanying images. A copy of the written consent is available for review by the Editor-in-Chief of this journal.
